# Impact of School-Based Interventions on Pediatric Obesity: A Systematic Review

**DOI:** 10.7759/cureus.43153

**Published:** 2023-08-08

**Authors:** Dhadon H Klein, Iman Mohamoud, Olawale O Olanisa, Panah Parab, Priti Chaudhary, Sonia Mukhtar, Ali Moradi, Athri Kodali, Chiugo Okoye, Ana P Arcia Franchini

**Affiliations:** 1 Internal Medicine, Saint James School of Medicine, St. Vincent, VCT; 2 Internal Medicine, California Institute of Behavioral Neurosciences & Psychology, Fairfield, USA; 3 Medicine, Semmelweis University, Budapest, HUN; 4 Research, California Institute of Behavioral Neurosciences & Psychology, Fairfield, USA

**Keywords:** behavioral change, motivation, nutritional change, physical activity, school-based interventions, pediatric obesity

## Abstract

Childhood obesity is a global public health problem with significant implications for the health and well-being of children. The prevalence of childhood obesity is increasing every decade, making it a recognized public health concern. This systematic review aims to explore and evaluate the impact of school-based interventions on reducing pediatric obesity among school-aged children. A systematic review of literature according to the recommendations of the Preferred Reporting Items for Systematic Reviews and Meta-Analyses (PRISMA) 2020 was conducted. Google Scholar, PubMed, and Cochrane were the databases used. After screening for bias, inclusion and exclusion criteria, and quality, 27 studies were included in the systematic review, and data were synthesized. The results show that physical activity reduces obesity and improves cardiovascular health. The nutritional change reduces the risk of obesity more than physical activity alone. When blended, the two provide the most benefits for participants. Motivation, self-efficacy, and behavioral change could help maintain the improvements. Schools should adopt a blend of physical activity and nutritional change to reduce prevent, reduce, and manage obesity.

## Introduction and background

Childhood obesity is a significant health concern characterized by excess body weight in children and adolescents. Typically, childhood obesity occurs when a child or adolescent’s weight exceeds the recommended range for their age and height, which in most instances is measured by using body mass index (BMI). A child is considered obese if they have a BMI higher than the 95th percentile [1]. The prevalence of childhood obesity is increasing rapidly; in 2022, according to the Centers for Disease Control and Prevention (CDC), in the United States, the prevalence of childhood obesity between 2017 and 2020 was 19.7%, affecting about 14.7 million children and adolescents. Among two- to five-year-olds, the prevalence was 12.7%, 20.7% among six- to eleven-year-olds, and 22.2% among twelve- to nineteen-year-olds. Childhood obesity, like general obesity, affects different ethnicities differently, leading to healthcare inequality. In the United States, Hispanic children have the highest prevalence at 26.2%, followed by non-Hispanic Black at 24.8%, then non-Hispanic White at 16.6%, and then non-Hispanic Asian children at 9% [2].

There are several components that can lead to childhood obesity, such as lifestyle, social and genetic factors. First, a poor diet; consumption of food with high-calorie value, such as sugary snacks, fast food, and soft drinks, contributes to weight gain. Second, a sedentary lifestyle; lack of physical exercise means a greater difference between caloric intake and expenditure. Television, video games, and smartphone over-engagement could predict childhood obesity because it reduces calorie expenditure and promotes inactivity. Third, genetic predisposition combined with environmental factors such as access to unhealthy foods and limited access to healthcare information and playgrounds [3]. Finally, socio-economic factors where children from lower economic backgrounds are at greater risk because of limited access to healthcare, physical activity tools, and healthy foods.

The ramifications of childhood obesity can extend beyond the early years and have a lasting impact on the child's life through adolescence and adulthood. The individual may be prone to developing psychological issues. Children with obesity are often at risk of stigmatization, low self-esteem, depression, and an increased risk of eating disorders. Although a causal link has not been established, research articles cited by Sahoo et al. show that samples of obese children have higher eating disorder traits, depressive symptoms, anxiety, emotional problems, body dissatisfaction, and low self-esteem compared to the control who are the children with BMI with the normal range [3]. Second, childhood obesity is associated with health issues. According to Balasundaram, obese children are at a higher risk of developing health complications, including type II diabetes, high blood pressure, heart diseases, sleep apnea, orthopedic problems, and asthma. These children are at risk of becoming obese adults at high risk for cardiovascular diseases and diabetes, major chronic diseases [1].

Due to the complexity of childhood obesity, effective interventions are needed at the school level to minimize the psychological and physical healthcare challenges these children persevere and to minimize the risk of developing chronic diseases at later stages of life. The United States loses $173 billion per year to obesity, $327 billion due to diabetes, and $217 billion due to cardiovascular diseases [4]. The diseases also lead to loss of life and productivity, not to mention that it leads to psychological and financial strain for the families of affected individuals. Timely interventions for childhood obesity can offset the psychological and financial strain for all parties involved.

To this end, school-based interventions have gained considerable attention as a potential strategy for preventing and reducing pediatric obesity. Schools are extremely crucial because they shape children’s behavior in the long term, and they increase access to the children creating a unique opportunity to promote healthy habits and lifestyles. As such, researchers have applied the appropriate interventions at schools and studied their effectiveness.

There is a gap in this research topic in that, to date, several studies have examined the impact of school-based interventions on pediatric obesity, but the findings have been inconsistent, and there is a need for a comprehensive synthesis of the available evidence. This systematic review aims to explore and evaluate the impact of school-based interventions on reducing pediatric obesity among school-aged children. The systematic review provides a narrative synthesis of the available evidence, examining the effectiveness of these interventions, exploring the research gaps, and reporting the potential of these interventions and how well they can be employed for the maximum benefit of obese children and other stakeholders. This paper is significant for the following parties. First, it will inform the policy-makers by proving them with some background information for decision-making. Second, it is a stepping stone for future researchers on the same topic and will shape community health providers’ decision-making.

## Review

Methods

Database and Search Strategy

The systematic review was conducted based on the recommendations of the Preferred Reporting Items for Systematic Reviews and Meta-Analyses (PRISMA) 2020. A systematic search of the PubMed, Google Scholar, and Cochrane databases was conducted and the studies were published between January 2018 to December 2022. Studies related to childhood obesity, impacts and outcomes, BMI, body fat, and school intervention or health services were searched. The detailed strategy, keywords, and Medical Subject Headings (MeSH) terms used are elaborated in Table 1 below. The titles and abstracts of all articles were independently screened.

**Table 1:** Keywords and MeSH terms used MeSH: Medical Subject Headings

Search terms	Database	Filters applied	Results
Impact OR outcome OR effect OR "Health Impact Assessment/statistics and numerical data" [Mesh]	PubMed	Clinical trial, randomized controlled trial, in the last five years, English	128,378 results
“pediatric obesity” OR “adolescent obesity” OR “BMI” OR “overweight” OR “body fat” OR ("Pediatric Obesity/classification" [Majr] OR "Pediatric Obesity/epidemiology" [Majr] OR "Pediatric Obesity/prevention and control" [Majr])	PubMed	Clinical trial, randomized controlled trial, in the last five years, English	7,050 results
“School-based intervention” OR ("School Health Services/classification" [Majr] OR "School Health Services/standards" [Majr] OR "School Health Services/statistics and numerical data" [Majr] OR "School Health Services/trends" [Majr])	PubMed	Clinical trial, randomized controlled trial, in the last five years, English	149 results
Impact OR outcome OR effect OR "Health Impact Assessment/statistics and numerical data" [Mesh] AND “pediatric obesity” OR “adolescent obesity” OR “BMI” OR “overweight” OR “body fat” OR ("Pediatric Obesity/classification" [Majr] OR "Pediatric Obesity/epidemiology"[Majr] OR "Pediatric Obesity/prevention and control" [Majr] ) AND “School-based intervention” OR ("School Health Services/classification" [Majr] OR "School Health Services/standards"[Majr] OR "School Health Services/statistics and numerical data" [Majr] OR "School Health Services/trends" [Majr])	PubMed	Clinical trial, randomized controlled trial, in the last five years, English	46 results
impact OR outcome AND pediatric obesity OR BMI OR body fat AND school intervention OR school program or school policy OR school health services	Google Scholar	January 2018 to December 2022	16,100 results
	Cochrane	January 2018 to December 2022	29,319 results

Inclusion Criteria 

The articles included in the systematic review are randomized and non-randomized controlled trials (RCTs), cross-sectional and quasi-experimental studies, and clinical trials conducted primarily in the school-based setting. Participants included students aged six to eighteen years. The studies conducted obesity interventions and reported outcomes of BMI or body fat. The interventions reported health and nutrition education or physical activity promotion as the primary intervention.

Exclusion Criteria

All clinical trials were excluded if they were not published in English, the setting was not at school, did not report outcomes on BMI or body fat, or primarily discussed the cost-effectiveness of interventions.

Results

Search Results

**Figure 1:**
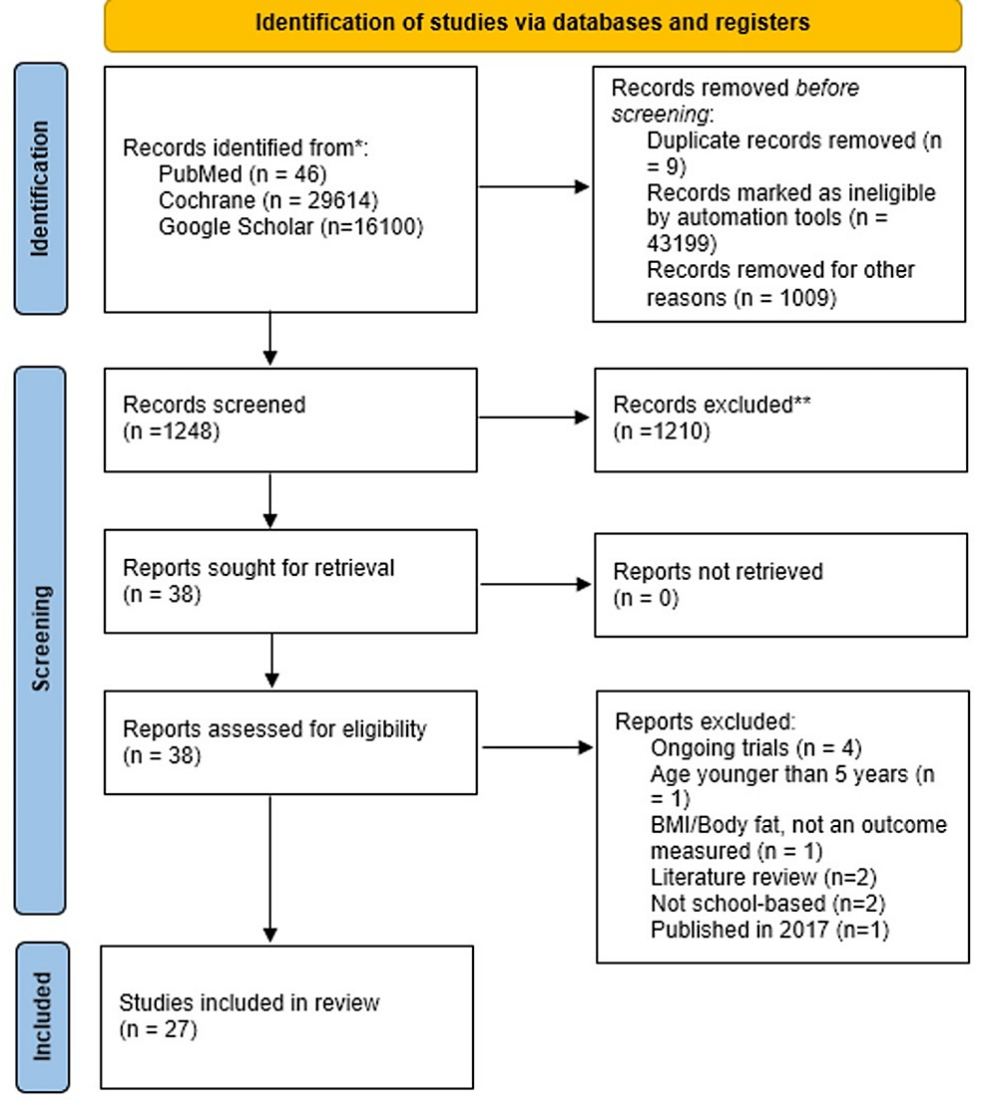
PRISMA flow diagram for the search process PRISMA: Preferred Reporting Items for Systematic Reviews and Meta-Analyses

Figure 1 displays the PRISMA flow diagram. The articles found in PubMed were 46, Cochrane 29614, and Google Scholar 16100. The duplicates were removed using the Endnote program, where nine records were removed. Automation tools marked 43199 records as ineligible for not being RCT, nRCTs, cross-sectional, and quasi-experimental studies clinical trials and 1009 records were removed after screening the titles based on the inclusion criteria. We screened 1248 records, and 1210 records were excluded. Of those, 38 records were sought to be retrieved, and all of them were found, and assessed for eligibility, where four were excluded since they are ongoing, two were excluded for using children younger than five years and not using BMI nor body fat as an outcome measure, two were literature reviews, two were not school-based, and one was published in 2017. The rest, 27, were included in a systematic review after being assessed for eligibility (Table 2).

**Table 2:** Critical appraisal for included studies RCT: randomized controlled trial, CASP: Critical Appraisal Skills Programme

Article title	Author	Type of study	Quality assessment tool used	Quality score					
Effectiveness of a childhood obesity prevention program delivered through schools, targeting 6 and 7 year olds: cluster randomized controlled trial (WAVES study)	Adab et al. [5]	RCT	CASP	10/12					83%
Frequency of school‐based intervention needed to improve weight outcomes of Mexican‐American adolescents with overweight or obesity: a randomized controlled trial	Arlinghaus et al. [6]	Open label-RCT	CASP	10/12				83%	
Assessment of the Efficacy of Physical Activity Level and Lifestyle Behavior Interventions Applying Social Cognitive Theory for Overweight and Obese Girl Adolescents	Bagherniya et al. [7]	RCT	CASP	9/12			75%		
Can School-Based Physical Activity Projects Such as Skipping Hearts Have a Long-Term Impact on Health and Health Behavior?	Baumgartner et al. [8]	Non-randomized controlled longitudinal trial	CASP	10/12			83%		
School-based gardening, cooking, and nutrition intervention increased vegetable intake but did not reduce BMI: Texas sprouts - a cluster randomized controlled trial	Davis et al. [9]	RCT	CASP	10/12		83%			
Effects of a physical activity intervention on schoolchildren's fitness	Di Maglie et al. [10]	RCT	CASP	11/12	92%				
Effect of High-Intensity Interval Training on Fitness, Fat Mass and Cardiometabolic Biomarkers in Children with Obesity: A Randomised Controlled Trial	Dias et al. [11]	RCT	CASP	9/12	75%				
Anthropometric outcomes of a motivational interviewing school-based randomized trial involving adolescents with overweight	Freira et al. [12]	RCT	CASP	12/12	100%				
Impact of a Three-Year Obesity Prevention Study on Healthy Behaviors and BMI among Lebanese Schoolchildren: Findings from Ajyal Salima Program	Habib-Mourad et al. [13]	Stratified cluster randomized intervention	CASP	9/12			75%		
Implementing School-Based Policies to Prevent Obesity: Cluster Randomized Trial	Ickovics et al. [14]	Cluster randomized trial using 2x2 factorial design	CASP	9/12			75%		
Effects of a School-Based Pedometer Intervention in Adolescents: 1-Year Follow-Up of a Cluster-Randomized Controlled Trial	Isensee et al. [15]	Cluster-RCT	CASP	12/12			100%		
Impact Of The Healthy, Hunger-Free Kids Act On Obesity Trends	Kenney et al. [16]	Cross-sectional study	CASP	11/12			92%		
The Great-Child^TM^ Trial: A Quasi-Experimental Intervention on Whole Grains with Healthy Balanced Diet to Manage Childhood Obesity in Kuala Lumpur, Malaysia	Koo et al. [17]	Quasi-experimental study	CASP	12/12			100%		
Cooking and Gardening Behaviors and Improvements in Dietary Intake in Hispanic/Latino Youth	Landry et al. [18]	RCT	CASP	10/12			83%		
Effectiveness of the Healthy Lifestyles Programme (HeLP) to prevent obesity in UK primary-school children: a cluster randomised controlled trial	Lloyd et al. [19]	Cluster-RCT	CASP	9/12			75%		
Differential effects of a school‐based obesity prevention program: A cluster randomized trial	Nickel et al. [20]	Cluster-RCT	CASP	12/12			100%		
Effect of school-based interventions on body composition of grade-4 children from lower socioeconomic communities in Gqeberha, South Afric	Nqweniso et al. [21]	RCT	CASP	11/12			92%		
Preliminary Results of the Planet Nutrition Program on Obesity Parameters in Mexican Schoolchildren: Pilot Single-School Randomized Controlled Trial	Ramírez-Rivera et al. [22]	RCT	CASP	12/12			100%		
Three-Year Follow-Up of the POIBA Intervention on Childhood Obesity: A Quasi-Experimental Study	Sánchez-Martínez et al. [23]	Quasi-experimental study	CASP	11/12			92%		
“FIFA 11 for Health” for Europe in the Faroe Islands: Effects on health markers and physical fitness in 10- to 12-year-old schoolchildren	Skoradal et al. [24]	RCT	CASP	12/12			100%		
The healthy body image (HBI) intervention: Effects of a school-based cluster-randomized controlled trial with 12-months follow-up	Sundgot-Borgen et al. [25]	Cluster-RCT	CASP	11/12			92%		
Effects of a practice-focused nutrition intervention in Hungarian adolescents	Takacs et al. [26]	RCT	CASP	9/12			75%		
Impacts of a School-Based Intervention That Incorporates Nutrition Education and a Supportive Healthy School Canteen Environment among Primary School Children in Malaysia	Teo et al. [27]	Quasi-experimental study	CASP	10/12			83%		
Effectiveness of the “Planning Health in School” Programme on Children’s Nutritional Status	Vieira et al. [28]	Non-RCT	CASP	12/12			100%		
Effectiveness of national multicentric school-based health lifestyles intervention among Chinese children and adolescents on knowledge, belief, and practice toward obesity at individual, family and schools’ levels.	Wang X et al. [29]	RCT	CASP	10/12			92%		
Childhood obesity prevention through a community-based cluster randomized controlled physical activity intervention among schools in China: the health legacy project of the 2nd world summer youth Olympic Games (YOG-Obesity study).	Wang Z et al. [30]	Cluster-RCT	CASP	12/12			100%		
Cluster randomised trial of a school-community child health promotion and obesity prevention intervention: findings from the evaluation of fun ‘n healthy in Moreland!	Waters et al. [31]	Cluster RCT	CASP	11/12			92%		

Quality Assessment

The articles were screened independently according to the inclusion and exclusion criteria. To evaluate the methodological quality of the 27 individual studies, we employed the Critical Appraisal Skills Programme (CASP) checklist shown in Table 2. The CASP checklists allowed us to assess the risk of bias, validity, relevance, and applicability of the included studies. Additionally, to ensure transparent and comprehensive reporting of our systematic review, we followed the PRISMA guidelines. The PRISMA checklist guided us in reporting the necessary information, such as the study selection process, data extraction methods, and synthesis techniques. The combined use of CASP and PRISMA facilitated a rigorous evaluation of study quality and enhanced the transparency of this systematic review.

Data Extraction

The data were extracted from the articles and tabulated in Table 3 below. We organized it starting with the author, study design, and country. Second, age in years and sample size, intervention (i), and control (c), with there being multiple interventions in some articles. Third, the intervention characteristics, including the type and duration of the intervention and follow-up. Finally, the outcome measures include changes in BMI, body fat, or obesity. For data synthesis, the studies were grouped by the type of intervention and the outcome measured reported.

**Table 3:** Study description and results RCT: randomized control trial, BMI: body mass index, i: intervention group, c: control group, HIIT: high-intensity interval training, MICT: moderate-intensity continuous training, PA: physical activity

Author	Study design	Setting	Age (years)	Sample size	Intervention	Intervention duration	Follow-up duration	Primary outcome measures	Results
Adab et al. [5]	RCT	United Kingdom (UK)	5-6	i(n = 26 schools; n=1134 students) c(n = 28 schools; 1328 students)	30 minutes of moderate to vigorous physical activity every school day; encouraging healthy eating, cooking workshops	12 months	15 & 30 months & 39 months in first group	BMI, body fat %	Non-significant lower BMI Score in the intervention arm compared to the control. (mean difference −0.075 (95% confidence interval −0.183 to 0.033, P=0.18) at 15 months. At 30 months, Lower BMI Score, Insignificant −0.137 to 0.083, p=0.63). No statistically significant difference between the groups for other anthropometric, dietary, physical activity, or psychological measurements
Arlinghaus et al. [6]	Open-label-RCT	United States of America (USA)	11-12	One day per week (n = 59) Three days per week (n = 58) Five days per week (n = 63) Zero days c(n = 63)	Established efficacy zero (control), one, three, or five days per week (equating to 0, 40, 120, or 200 min of contact each week) 80% of intervention time on physical activity and 20% to nutrition, and behavioral modification	12 months	12 months	zBMI	A significant condition by time interaction was observed ((F = 9.42, P < .001). Receiving intervention 2/3 times per week significantly decreased zBMI than control (−0.19 zBMI units/y; 95% CI, −0.28 to −0.11; and −0.18 zBMI units/y; 95% CI, −0.27 to −0.10, both P < .001)
Bagherniya et al. [7]	RCT	Iran	12-16 (Females)	i(n = 104) c(n = 98)	Sessions of sport (each session 90 min) were held twice a week (60 sessions in 7 months) delivered by a specialist in physical education and sport workshops and interactive seminars twice a month for students (14 sessions)	Seven months	3.5 months & seven months	BMI	The subject’s mean BMI and Waist circumference was reduced in the intervention group from 29.47 (4.05) kg/m2 to 28.5 (4.35) kg/m2 and 89.65 (8.15) cm to 86.54 (9.76) cm. However, the changes were not statistically significant (p=0.127 and P=0.504, respectively)
Baumgartner et al. [8]	Non-RCT longitudinal trial	Germany	8-9	SH-B only the 90-min Basic-Workshop (n= 838) SH-CH Champion-Program in addition to the Basic-Workshop (n= 480) Control group, neither the Basic-Workshop nor the Champion-Program) (n= 344)	Skipping Hearts(SH) project, which aims to promoting physical activity and an active lifestyle through rope skipping during childhood; with a one-time 90-min Basic-Workshop and the subsequent Champion-Program with ten 45-min rope skipping lessons	Three years	Three years	BMI, body fat	Standard deviation of BMI and systolic blood pressure decreased insignificantly while the motor performance, physical fitness, subjective physical activity and screen-based media use increased. In all p>0.05
Davis et al. [9]	RCT	USA	8-11	i(n = 8 schools; 1711 students) c(n = 8 schools; 2528 students)	Gardens built at school and eighteen 1-hour lessons on gardening, nutrition & cooking	Nine months	Nine months	BMI	School-based gardening, nutrition, and cooking program did not reduce obesity markers or blood pressure. Gardening was significantly associated with increase in vegetable intake
DiMaglie et al. [10]	RCT	Italy	11-12	i(n = 80 students) c(n = 80 students)	Additional 40 min of physical activity 5-6 days per week	Six months	Six months	BMI	There was significant change in BMI in the intervention group (−2.4 ± 0.6 kg/m2) than the control group. The same is true for waist circumference, waist-to-height ratio, and physic fitness. Further, obesity decrease was significant, i.e., 17%
Dias et al. [11]	RCT	China	11-13	i(HIIT, n = 15) i(MICT, n = 15) c(n = 15).	3 groups: high-intensity interval training (HIIT), moderate-intensity continuous training (MICT), or non-exercising control	12 weeks	12 weeks	BMI, body fat mass	There was an increased cardiorespiratory fitness health, but the obesity measures did not change significantly
Freira et al. [12]	RCT	Portugal	14-19	i(n = 46) c(n = 51)	Everyone received diet counseling and an hour of moderate to vigorous exercise daily. Intervention group received motivational interviewing, while the control group received conventional counseling	Three 30-min sessions every three months	Nine months	BMI, abdominal circumference, body fat %, muscle mass %	Significant positive changes in anthropometric variables including BMI z-score, abdominal circumference, percentage of fat mass, percentage of muscular mass, systolic and diastolic blood pressure were recorded in 6 months after 3 sessions. Conventional intervention group did not have favorable results
Habib-Mourad et al. [13]	Stratified cluster RCT	Lebanon	8-12	i(n = 698) c(n = 541)	Promotion of healthy eating and an active lifestyle; 15 min of discussion, information and interaction on a topic followed by 30 min of activity: game and/or food preparation; family involvement; food service intervention targeting the school shops and the lunch boxes sent by the family	Two years	Two years & three years	BMI	The number of students reporting dietary changes in the intervention group significantly increased compared to the control. Students in the intervention group were less likely to be overweight in public schools
Ickovics et al. [14]	Cluster RCT using 2 × 2 factorial design	USA	10-11	Nutrition only (n = 169) PA only (n = 221) Nutrition & PA (n = 195) Delayed (n = 134)	Four groups: nutrition only, physical activity only, nutrition and physical activity (dual), or delayed	Three years	One, two, and three years	BMI	Intervention schools had a healthier BMI trajectory over time (F=3.20, p=0.02). A greater magnitude was observed with time with the more significant results recorded at the third year. There were improvements in healthy eating
Isensee et al. [15]	RCT	Germany	12-15	i(n=790) c(n=506)	Measure activity through a pedometer with weekly class competitions	12 weeks	12 weeks & one year	BMI	Significant results were observed in Body fat and waist ratio. No significant results were obtained on BMI, but cardiorespiratory fitness and physical activity increased but did not meet significance
Kenney et al. [16]	Cross-sectional study	USA	10-17	n = 173,013	Policies to improve the nutritional quality of food and beverages	One year	One year	BMI	The results showed significant reduction for obesity risk for children in poverty where the policy reduced the expected prevalence of obesity among children living in poverty in 2018 by 47%
Koo et al. [17]	Quasi-experimental study	Malaysia	9-11	i(n = 31) c(n = 32)	six 30-min nutrition education lessons, school delivery of wholegrain food on a daily basis, and parents attended a 1-hour individual diet counseling	12 weeks	Three and nine months	BMI, body fat, waist circumference	Intervention group showed significantly lower zBMI scores (−0.12; 95% CI: −0.21, −0.03; p = 0.009), body fat percentage (weighted difference: −2.6%; 95% CI: −3.7, −1.5; p < 0.001) and waist circumference (weighted difference: −2.4 cm; 95% CI: −3.8, −1.0; p = 0.001) than the control group. Time was a factor since the body fat percentage and waist circumference was lower at three months
Landry et al. [18]	RCT	USA	10-11	i(n = 204) c(n = 166)	45 minutes of cooking and nutrition curriculum in addition to 45 minutes of gardening curriculum afterschool	12 weeks	12 weeks	BMI, waist circumference	Intervention group showed significantly greater reductions in BMI z-scores (−0.1 vs. −0.04, respectively; p=0.01) and WC (−1.2 vs. 0.1cm; p<0.001). Fewer had the metabolic syndrome (MetSyn) after the intervention than before, while controls with MetSyn increased. Increase in cooking behaviors significantly predicted increase in fiber intake (p=0.004) and increase in vegetable intake (p=0.03). Increase in gardening behavior was highly associated with dietary fiber intake (p=0.02)
Lloyd et al. [19]	RCT	UK	9-10	i(n = 676) c(n = 648)	Physical activity workshops, education sessions delivered by teachers with short homework tasks, drama sessions, and setting goals to modify behavior	36 weeks	18 and 24 months	BMI, body fat	A minor reduction of BMI scores was observed in the intervention groups i.e., -0·02 (95% CI -0·09 to 0·05), p=0·57 after one year. This is not statistically significant
Nickel et al. [20]	RCT	Canada	6-12	i(n = 340) c(n = 347)	Weekly lessons on healthy eating, physical activity and self‐efficacy and two 30‐min structured physical activity sessions	21 weeks	21 weeks	BMI, waist circumference	There was significantly greater reduction in waist circumference among the participants than control. There were improvements in dietary intake, healthy-living knowledge and self-efficacy. The effects were more favorable in boys and urban dwellers
Nqweniso et al. [21]	RCT	South Africa	8-11	School 1: PE i(n= 90) c(n=113) School 2: PE, H&H (n=99) c(n=97) School 3: H&H, NS i(n=92) c(n=151) School 4: PE, H&H, NS i(n=170) c(n=86)	All students took deworming meds. Interventions included: two weekly 40-min physical education (PE) lessons, six 45-min health and hygiene (H&H) lessons, or six 45-min nutrition education & supplement (NS)	10 weeks	10 weeks	BMI, body fat %	In normal-weight children and obese children, physical activity alone or coupled with health education mitigated the increase in body fat
Ramirez-Rivera et al. [22]	RCT	Mexico	9-12	i(n = 21) c(n = 20)	Two 1-hour nutrition education classes per week and three 1-hour PA sessions per week. Control group 1 hour of general nutrition recommendations at the end of the study	Nine weeks	Nine and 23 weeks	BMI, body fat %, waist circumference	Significant differences were observed in body fat percentage, waist circumference, and nutrition knowledge. Summer holidays negatively affected the zBMI Score. BMI change was there but insignificant
Sánchez-Martínez et al. [23]	Quasi-experimental study	Spain	8-9	i(n = 1633) c(n = 1991)	Nine sessions: 58 activities, classified into 3 modules. Module 1 worked on growth (weight and height) and body image assessment. Module 2 focused on food and nutrition. Module 3 worked on physical activity and rest	Nine months	One year & three years	Weight, height, triceps skinfold thickness, and waist and hip circumference , then BMI	No differences were found for BMI in the effect of the intervention between the IG and the CG. Children in the intervention group had significantly better healthy habits regarding physical activity(16.2% IG vs. 11.9% CG; p = 0.012) and nutrition lobal score (63.9% IG vs. 58.5% CG; p = 0.025). The trend loses significance at three years but continues to be positive
Skoradal et al. [24]	RCT	Faroe Islands	10-12	i(n = 292) c(n = 100)	Education program based on football practice and health education. two weekly sessions (45 minutes each)	11 weeks	11 weeks	Body fat %, lean body mass	Systolic blood pressure reduced significantly in the intervention group. Lean body mass, postural balance and horizontal jump performance increased significantly. There was a significant BMI reduction within the intervention group
Sundgot-Borgen et al. [25]	Cluster RCT	Norway	16-17	i(n = 1499) c(n = 947)	HBI intervention focuses on positive embodiment and health-related quality of life and employs an interactive educational approach with a 90 minute interactive workshop every three weeks	Three months	Three and 12 months	BMI	There was favorable immediate change in body image for girls and health related quality of life. The change for boys was however weak
Takacs, Martos and Kovacs [26]	RCT	Hungary	11-13	i(n = 112) c(n = 112)	Weekly classroom-based education (25 to 45 minutes long); five sessions of after-school cooking classes	Nine months	Nine and 12 months	Body fat	There were slight improvements in dietary knowledge and habits from baseline to post intervention. Aerobic fitness increased significantly among the intervention group and not control. Waist circumference increased in control but not in IG, especially in summer. Other Anthropometric measures remained constant
Teo et al. [27]	Quasi-experimental study	Malaysia	7-11	i(n = 251) c(n = 272)	Nutrition education six hours per month and a healthy school canteen environment	Three months	Three and six months	BMI	The intervention showed significantly increased frequency for lunch, breakfast, and dinner consumption, physical activity, and cognitive performance than the Control. The BMI for the IG was significantly lower than control
Vieira, Teixeira and Carvalho [28]	Non-RCT	Portugal	10-14	i(n = 219) c(n = 230)	Promote healthy eating and active living	Nine months	Nine months	BMI, waist circumference, waist-to-height ratio	The intervention group significantly reduced the waist circumference while the control significantly increased. The IG group was significantly taller and had a reduced waist-to-height ratio than the control group. There were significant improvements in physical activity time in the IG
Wang et al. [29]	Cluster RCT	China	6-15	i(n = 27,477) c(n = 30,997)	Create a supportive school and family environment; promote health lifestyles education and related compulsory physical activities; instruct and promote school physical education; self-monitor obesity related behaviors	Nine months	Nine months	BMI	The intervention group had higher obesity related-knowledge correctness, and beliefs and had assumed healthy habits compared to the control group. The prevalence of obesity was lower in intervention than control groups
Wang et al. [30]	RCT	China	8-9 11-12	i(n = 5400) c(n = 4691)	45 min health education monthly; school environment support, family involvement; fun programs/events	10 months	10 months	BMI	Physical activity significantly increased in the Intervention group while it reduced in the CG. The BMI and zBMI scores in the intervention group were significantly lower. Further, the group was less likely to be obese
Waters et al. [31]	RCT	Australia	4-13	i(n = 1594) c(n = 1628)	Increasing fruit, vegetable and water consumption; increasing physical activity; encouraging positive self-esteem in children	3.5 years	Four to five years	BMI	There were no significant changes in the BMI score in the intervention group. However, they were more likely to consume more daily fruit serves and water and less likely to have fruit cordial

Discussion

Encouraging Healthy Eating

Healthy eating is vital in preventing and managing obesity. A nutritious diet contributes to weight control, improves overall health, and reduces the risk of obesity-related complications. A healthy diet works by controlling the caloric intake, hence creating a caloric balance, provides essential nutrients that support overall health through weight management, helps in understanding the significance of portion sizes and therefore encourages healthy eating patterns and servings, reduces the intake of added sugar and processed food, not to mention it helps in the balance of macronutrients. In this systematic review, seventeen studies included nutrition, dietary change, and education. Fourteen of the seventeen studies recorded statistically significant results in at least one of the measures taken, including a reduction in waist size, reduction in the percentage of fat, reduced zBMI, or improved diet and nutritional knowledge. 

Administering dietary change lessons with a follow-up, such as lessons on cooking or the provision of food, significantly reduces obesity. Arlinghaus et al. included 20% nutritional change education in their study and reported a significant decrease in zBMI [6], which was more significant at the end of the intervention, 12 months. Likewise, three studies featured dietary counseling [12, 22, 27], such as lessons in healthy eating, where Nickel et al. offered 21 lessons making this the priority compared to physical activity and self-efficacy [20]. These studies reported a great reduction in waist circumference, BMI Z-score, percentage of fat mass, and improvements in dietary intake.

Nutritional knowledge helps foster personal responsibility vis-à-vis healthy eating. While diet programs can help the child reduce their anthropometric measures, they might lose effectiveness over time when not linked with nutritional knowledge. Nutritional knowledge helps children make informed decisions about their food intake and adopt healthier eating patterns. Education also helps in portion control and helps in reducing the excess calories that could be stored as fat by maintaining a balance between caloric intake and expenditure. This explains the favorable outcomes shown by lessons on dietary change [13, 17, 22].

While nutrition knowledge can be sufficient, some children need more tools and modeling, such as lessons on cooking, correct portions, and a balanced diet. To some, poverty can hinder practicing the changes highlighted during the lessons. Koo et al. offered nutritional education lessons and delivered whole-grain food daily for 12 weeks to fill this gap [17]. It is worth noting that this was the only intervention offered in this program. This program yielded a reduced zBMI score, reduced body fat percentage, and reduced waist circumference, where each effect increased as time elapsed [17]. Takacs et al. offered education and after-school cooking classes, and as a result, while the control gained significant waist circumference in summer, this group reduced [26]. However, this was the only significant change in anthropometric measures. Vieira et al. enhanced the consumption of vegetables, decreased sugary food, and consumption of high-fat and energy-dense food consumption, and reported decreased waist circumference [28]. These programs might be more effective on children since, at times, they need modeling. It is also effective for children coming from poor backgrounds.

The systematic review also reveals that while not all nutrition interventions were statistically significant in the anthropometric measures, they all positively impacted the children in diverse ways. For example, Habib-Mourad et al. improved dietary change and reduced the risk of obesity, in the intervention group compared to the control group [13]. Likewise, Ickovics et al. reported a healthier BMI trajectory as time elapsed and a decrease in the consumption of unhealthy foods [14]. Kenney et al. reported that with the passage of the Healthy, Hunger-Free Kids Act the odds of obesity reduce by 9% annually for youth in poverty [16]. Davis et al. and Landry et al. recorded more vegetable and dietary fiber intake [9,18]. 

These studies show that even when not statistically significant, nutritional interventions reduce the risk of obesity. Increasing vegetable and fruit intake with high fiber, essential nutrients, and low calories promotes satiety, provides vitamins, and helps manage caloric intake reducing the risk of obesity. Whole grains such as wheat, brown rice, oats, and quinoa boost the fiber and nutrient intake, which helps the children feel full for longer, reducing their yearning for food and the risk of obesity by extension [18]. Water helps keep one hydrated and helps avoid caloric consumption through soft drinks [31]. 

Physical Activity Effect on Pediatric Obesity

Physical activity is crucial in the prevention and management of obesity. The conventional knowledge is that through moderated physical activity, there is an increase in energy expenditure, creating an energy deficit critical for weight loss. However, the importance of physical activity is more than an energy deficit. In this systematic review, seventeen studies included physical activity in practice or education. However, statistical significance when the anthropometric was achieved in only nine of them. Excluding studies that incorporated nutritional interventions, only four studies reached statistical significance. Positive effects for physical activity were closely associated with higher than four months of physical activity and moderate to intensive physical exercise.

Four studies attained statistical significance without including dietary changes. Di Maglie et al. found a statistically significant reduction in BMI, waist circumference, waist-to-height ratio, and physical fitness for those who had forty minutes of physical training five-six days a week for six months [10]. Wang et al., Skoradal et al., and Isensee et al. all reported improved anthropometric measures such as reduced decreased body fat and waist ratio, increased lean mass, and reduced BMI and zBMI measures [30, 24, 15]. Most of them featured over forty-five minutes of physical activity. 

The positive effects can be explained by the following. First, an increased expenditure allows the body to burn calories. Second, it increases metabolism during the intervention and afterward, making the body burn calories even when not exercising. Third, engaging in strength training exercises not only aids in reducing body fat but also plays a vital role in promoting the growth and preservation of lean muscle mass, emphasizing the significance of prioritizing muscle gain over fat loss. [24]. Fourth, regular exercise regulates hunger hormones such as ghrelin and leptin, contributing to weight management. Further, it helps in maintaining weight loss and sustaining intermediate results. As such, despite the lower significance achievement rates, the importance of physical activity cannot be understated.

Five studies coupled physical activity with nutritional health lessons and attained significance. Arlinghaus et al., Freira et al., Nickel et al., Ramírez-Rivera et al., and Vieira et al. found statistically significant results [6, 12, 20, 22, 28]. The studies take longer than nine months, and the intensity of the physical exercises is comparable to those with physical exercise as an intervention only. As the studies show, coupling dietary change with exercise is more associated with significant results than physical activity in isolation.

Coupling dietary change with physical activity works for several reasons. First, while physical activity maximizes caloric expenditure, diet change ensures the deficit is not filled by more caloric intake. This favors weight loss. Second, a healthy diet is needed to maintain lean and muscle mass after exercise. Third, coupling physical activity and dietary change ensures the children do not overdo the diet. This is because physical activity reduces appetite to some extent. 

The studies that featured non-intensive physical activity in terms of the session or the program length were more likely to have insignificant positive effects. Adab et al., Bagherniya et al., Baumgartner et al., and Sánchez-Martínez et al. all feature sessions shorter than forty minutes or programs shorter than nine months, light exercises or all [5, 7, 8, 23]. This points toward the argument that the length of the exercise sessions and the program influences the effectiveness of an obesity program. There is a need for research testing this hypothesis since none of the studies answers the question.

Another observation was made for physical activity. Physical activation was the only intervention that was associated with improved cardiac health, including diastolic and systolic blood pressure and cardiorespiratory fitness. Isensee et al., Freira et al., Dias et al., and Baumgartner et al. all reported significant improvements in cardiovascular health [15, 12, 11, 8]. Obesity is more lethal when coupled with cardiovascular diseases, especially in the latter stages of life. These studies illustrate that physical activity significantly reduces this chance by increasing cardiovascular health [32]. 

Behavioral Change

There were only four studies that used behavioral modification as an intervention. None of the studies involved behavioral modification without additional intervention. Arlinghaus et al. and Vieira et al. were the only studies that included behavioral change and got significant results [6,28]. However, it should be noted that behavioral change is hugely included in diet and physical activity modification which have been discussed in the earlier sections. Nonetheless, behavioral change as an intervention includes other strategies such as goal setting, where the children are shown how to create attainable goals and work toward them, problem-solving and coping mechanisms, where children are shown how to overcome adversity and challenges that could lead to unhealthy eating patterns and consequently obesity. Furthermore, children learn long-term maintenance of the behavioral change adopted in other programs and learn how to engage with a supportive social network and self-monitor. As such, the importance of behavioral change in this systematic review cannot be established. However, behavioral change could help reduce the emotional, psychological, and environmental factors that are likely to lead to an eating disorder. Moreover, after achieving intermediate positive results, behavioral change lessons such as goal setting, self-monitoring, and long-term maintenance could be critical in sustaining them. 

Motivation and Self Efficacy

Self-efficacy and motivation are key factors in mediating behavioral change through physical activity, dietary change, long-term maintenance of the change, problem-solving, and self-monitoring. In the systematic review, four studies focused on this aspect. Freira et al. recorded significant changes in BMI z-score, abdominal circumference, fat mass reduction, and increase in the percentage of muscular mass [12]. Nickel et al. found favorable results in reducing waist circumference [20]. Waters et al. did not find significant results but observed improvements in healthy eating [31]. Finally, Sundgot-Borgen et al. found that girls were likelier to have a positive body image which is critical in their psychosocial health. By using the Healthy Body Image intervention female students were more likely to have a positive body image leading to body acceptance which may transform into better psychological well-being. Positive correlations with measures of body esteem were found to be negatively associated with objectified body consciousness, eating problems, alexithymia, and depression [25].

External factors such as rewards, recognition, or support from others influence motivation. For example, participating in a weight loss program, joining a support group, or receiving praise and encouragement from loved ones can boost motivation and help individuals stay on track with their weight management efforts. On the other hand, self-efficacy can be built through successful experiences, modeling, verbal persuasion, and managing emotional and physiological states. Setting realistic goals, breaking them down into smaller achievable steps, and celebrating small victories can enhance self-efficacy and boost confidence in children's ability to address obesity effectively. By fostering motivation and building self-efficacy, individuals are more likely to engage in and sustain the behavior changes necessary for addressing obesity.

Limitations and suggestions

Despite the valuable insights provided by this systematic review, there are several limitations that need to be acknowledged. First, the included studies varied in terms of design, sample size, duration of intervention, and outcome measures, which may introduce heterogeneity and limit the generalizability of the findings. The lack of standardization across studies makes it difficult to directly compare the results and draw definitive conclusions. There is a need for standardized protocols and outcome measures across studies to facilitate comparability and enhance the strength of evidence. Researchers should consider using validated tools and guidelines, such as the WHO's Health Promoting Schools framework, to ensure consistency and improve the quality of research in this field. Second, the majority of the studies relied on self-reported data, which may be subject to recall bias and social desirability bias. Participants might overestimate their level of physical activity or underestimate their dietary intake, leading to inaccurate measurements. Future studies should consider using objective measures, such as accelerometers or dietary logs, to obtain more reliable data. Third, the duration of the interventions varied widely, ranging from a few weeks to several months. Short-term interventions may not fully capture the long-term effects of school-based interventions on pediatric obesity. Longer follow-up periods are needed to assess the sustainability of the interventions and their impact on obesity prevention and management over time. Tracking participants' progress beyond the intervention period can provide valuable insights into the maintenance of healthy habits and inform strategies for long-term weight management. Fourth, the studies included in the review focused primarily on physical activity and nutritional change as interventions. While these two components are essential, other factors such as psychological and social determinants of behavior change were not adequately addressed. Future research should explore the incorporation of behavioral change strategies, such as goal setting, problem-solving, and social support, to enhance the effectiveness of school-based interventions. Lastly, future research should explore the potential benefits of involving multiple stakeholders, including parents, teachers, and healthcare professionals, in the design and implementation of school-based interventions. Collaboration among these key stakeholders can create a supportive environment for behavior change and promote a comprehensive approach to pediatric obesity prevention and management.

## Conclusions

We established that nutritional change through education alone or through education coupled with further action, such as lessons on cooking or provision of food, is the most effective strategy when each intervention is taken alone. It is followed by physical activity that exceeds forty minutes for over nine months. Conventional wisdom supports behavioral change, motivation, and self-efficacy, but the evidence from the systematic review fails to support them in isolation. However, each of them shows some effectiveness when coupled with other interventions such as dietary change or physical activity. The intervention blend with the highest likelihood of influencing obesity measures is coupling physical activity and nutritional change education. These findings have implications for various stakeholders, including policy-makers, school administrators, community health officials, and parents among others. This paper recommends the adoption of a blend of physical activity and nutritional change in schools. The intervention must take longer than a year to determine its effectiveness with regard to obesity.

Future work on this topic should focus on addressing the effect of physical activity on children’s mental health. While it is known that imposed physical activity takes a mental toll on children, none of these studies directly addressed it or the children’s perception of physical activity in the latter stages of life. A longitudinal study on this topic could be informative. Further, more studies across populations and regions are needed to confirm the finding that coupling physical activity with dietary change has the highest level of significance. In conclusion, while school-based interventions show promise in reducing pediatric obesity, there are limitations that need to be addressed to enhance the quality and effectiveness of these interventions. By addressing the identified limitations and implementing the suggested strategies, future research and interventions can contribute to the development of evidence-based approaches to combat pediatric obesity and improve the health and well-being of children.
