# Correction: Impact of School-Based Interventions on Pediatric Obesity: A Systematic Review

**DOI:** 10.7759/cureus.c179

**Published:** 2024-05-24

**Authors:** Dhadon H Klein, Iman Mohamoud, Olawale O Olanisa, Panah Parab, Priti Chaudhary, Sonia Mukhtar, Ali Moradi, Athri Kodali, Chiugo Okoye, Ana P Arcia Franchini

**Affiliations:** 1 Internal Medicine, Saint James School of Medicine, St. Vincent, VCT; 2 Internal Medicine, California Institute of Behavioral Neurosciences & Psychology, Fairfield, USA; 3 Medicine, Semmelweis University, Budapest, HUN; 4 Research, California Institute of Behavioral Neurosciences & Psychology, Fairfield, USA

 

This article has been corrected at the request of the first author to change his affiliation as follows:

Dhadon H. Klein Original: Internal Medicine, Saint James School of Medicine, Park Ridge, USA Corrected: Internal Medicine, Saint James School of Medicine, St. Vincent, VCT

The authors deeply regret that these errors were not identified and addressed prior to publication.

 

